# *De novo* Assembly and Transcriptome Characterization of *Opisthopappus* (Asteraceae) for Population Differentiation and Adaption

**DOI:** 10.3389/fgene.2018.00371

**Published:** 2018-09-19

**Authors:** Min Chai, Shengnan Wang, Juan He, Wei Chen, Zelu Fan, Jia Li, Yiling Wang

**Affiliations:** ^1^College of Life Science, Shanxi Normal University, Linfen, China; ^2^Institute of Animal Science, Chinese Academy of Agricultural Sciences, Beijing, China

**Keywords:** *Opisthopappus* Shih, transcriptome, RNA-Seq, gene expression difference, population differentiation, population adaption

## Abstract

*Opisthopappus* Shih (Asteraceae), an endangered genus endemic to the Taihang Mountains of China, is a high-value ornamental and medicinal plant consisting of two species, *Opisthopappus longilobus* shih and *Opisthopappus taihangensis* (Ling) Shih. However, the evolutionary relationships and the taxonomic characteristics between the two species remain unknown. In this study, high-throughput transcriptome sequencing was used to analyze the differential metabolic activity and gene expression and screened special molecular markers for exploring the genetic variation and species differentiation in *Opisthopappus* Shih. The results showed that 33,974 unigenes with an average size of 801 bp were obtained with optimization of *de novo* assembly. The comprehensive functional annotation based on Gene Ontology (GO), Cluster of Orthologous Group (COG) and Kyoto Encyclopedia of Genes and Genomes pathway database (KEGG) revealed that these unigenes were mainly related to many physiological, metabolic, and molecular processes. Furthermore, the comparative transcriptome analysis indicated that 3,410 differentially expressed genes were mainly involved in lipid, carbohydrate and amino acid metabolism, xenobiotics biodegradation and metabolism as well as environment adaptation via KEGG. Such as the *CYP710A*, *GST*, *HSP90A* and so on, could be the potential candidate genes for further investigating the molecular mechanism of physiological variations between *O. taihangensis* and *O. longilobus*. In addition, the potential 71,804 high quality single nucleotide polymorphisms (SNPs) and 1,444 simple sequence repeats (SSRs) were estimated. Based on the predicted SNP, we have developed eight SNP markers for population genetic analysis in *Opisthopappus* Shih. A significantly high level of genetic differentiation between the populations of *O. longilobus* and *O. taihangensis* were found, and they were clearly grouped into two distinct genetic clusters. These results conformed to the record of Flora Reipublicae Popularis Sinicae (FRPS) and unsupported the taxonomic status in the Flora of China. The transcriptome analysis of *Opisthopappus* Shih can contribute to in-depth exploring of internal mechanisms in species variation and differentiation based on molecular evidence. With the rich and valuable data resources, the more novel structural, functional, and comparative genomic studies will provide comprehensive insights into the evolutionary relationships between *O. taihangensis* and *O. longilobus*.

## Introduction

The genus *Opisthopappus* Shih belonging to the family Asteraceae, was generally considered as consisting of two species, *Opisthopappus longilobus* shih and *Opisthopappus taihangensis* (Ling) Shih (Shih, 1979). Being a perennial herbaceous, this genus is endemically distributed in China and is mainly restricted to the Taihang Mountains across the provinces of Shanxi, Hebei, and Henan. It naturally grows on cliff cracks, rock gaps in open forests below cliffs and infertile soil at an elevation of 1,000 m. The desirable traits of *Opisthopappus* Shih including drought tolerance and leanness-resistant could provide an extensive reservoir of genetic variation for gene excavation and function. Additionally, large white-flowered *O. longilobus* and *O. taihangensis* with remarkably high ornamental and medical values are used as potential genetic resource for chrysanthemum improvement and modern breeding (Shih and Fu, 1983; Yang et al., 2010; Zhao et al., 2010; Tang et al., 2012). However, the distribution range of *O. longilobus* and *O. taihangensis* is continuously decreasing due to the changes in their habitats and man-made damage, and they have been listed among the Class II State-Protected Endangered Plant Species, which means that relevant studies are urgently needed to carry out and offer effective strategies and potential candidate genes for the protection and utilization of endangered and endemic species (Wang and Xie, 2009).

At present, evolutionary relationships and taxonomic status of species within *Opisthopappus* Shih have always been debated and remain inconclusive, and some researchers believe that *O. longilobus* and *O. taihangensis* are confirmed as distinct species based on their morphological characteristics (Hu, 2008; Xu et al., 2011). Specifically, strict leaf morphological characteristics are often used as a fundamental parameter to distinguish them. *O. longilobus* has a smooth and sub cylindrical, whereas *O. taihangensis* is pubescent on both surfaces of the leaf blade. Moreover, *O. longilobus* pinnatifid has a pair of bracteal leaf below the involucres, whereas *O. taihangensis* bipinnatifid has none (Wang et al., 1978; Ding and Wang, 1997). The comparison of the biological characteristics of *O. longilobus* and *O. taihangensis* in its natural habitat and artificial population showed their stable inheritable character and significant difference in the morphologic features of leaf and plant type (Hu, 2008). Further research on the pollen morphology of *Opisthopappus* Shih, the results showed obvious differences in the pollen size, aperture, extine thickness and ornamentation, and *O. longilobus* was more highly evolved than *O. taihangensis* (Gao et al., 2011; Jia and Wang, 2015).

Additionally, with the rapid development of various molecular markers such as RFLP, RAPD, ISSR, simple sequence repeat (SSR), SR, AFLP and single nucleotide polymorphism (SNP), the genetic diversity analyses of *Opisthopappus* Shih populations were carried out to obtain more comprehensive information and data about genetic differentiation and variation. The results revealed abundant genetic diversity in populations but low level of genetic differentiation among populations and confirmed that the genetic difference of the species mainly existed within populations (Guo et al., 2013). Meanwhile, the cluster analysis based on the genetic distance showed that *O. longilobus* populations were not monophyletic group, and the remaining populations of *O. longilobus* were gathered with *O. taihangensis* populations, which indicated that gene exchange or interspecific hybridization might occur between the two species (Wang et al., 2015). Therefore, considering that the morphological variations of the two species were probably affected by the regional environmental condition, they might be classified as one species. Moreover, it was speculated that a transitional species of the genus of *Opisthopappus* Shih might exist as the third species in overlapped areas, whereas *O. longilobus* and *O. taihangensis* were two separate species located in a different geographical region.

However, no clear and systematic evidence unveils interspecific differences and confirms the taxonomical position between *O. longilobus* and *O. taihangensis*. A simple, rapid and transcriptome analysis approach is a solution for large genomes that enable the reduction of genome sequence complexity by focusing on genic regions. Moreover transcriptome sequencing is a powerful tool for identifying a larger scale of differentially expressed genes involved in the morphological, physiological and metabolic activity and developing a great deal of useful molecular markers distributed throughout the genome, such as SSRs and SNPs (Barker et al., 2009; Mckain et al., 2012; Wen et al., 2013).

**FIGURE 1:**
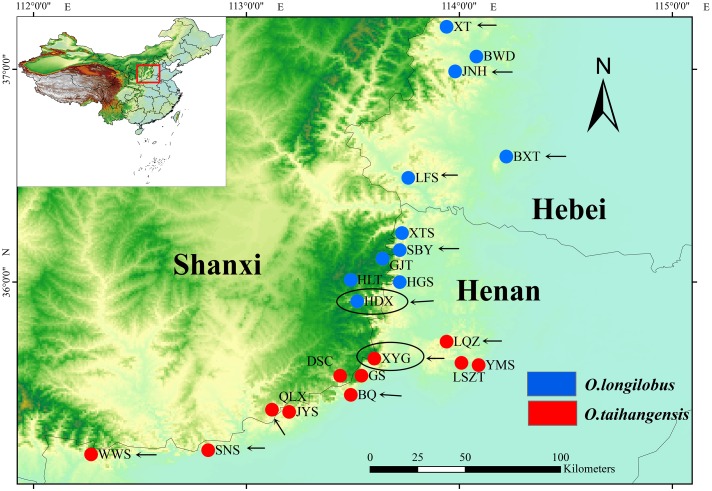
Distribution of *Opisthopappus* Shih populations. The blue dot represents the distribution of O. *longilobus*. The red dot represents the distribution of O. *taihangensis*. The black oval represents the sampling sites of transcriptome sequencing. The black arrow represents the sampling sites of SNP analysis.

Therefore, in this study, based on the premise of the existence of two putative species within *Opisthopappus* Shih, the XYG population of *O. taihangensis* and HDX population of *O. longilobus* growing in similar geographic and climate conditions were carefully chosen for comparative transcriptome analysis (**Figure 1** and **Table 1**). Our main objectives were to (1) compare the morphological, physiological and metabolic activity and further identify the differential functional genes involved in the above pathway, (2) screen, develop, and validate special molecular markers, which are beneficial to explore the molecular mechanism and evidence of phenotypic and genetic variation and species differentiation, and for in-depth study on population genetics and phylogeography of *Opisthopappus* Shih.

**Table 1:** Location of the natural populations.

Population	Species name	Location	Longitude (E) (°)	Latitude (N) (°)	Altitude (m)
XT	*O. longilobus*	Xingtaidaxiagu, Hebei	113.94	37.20	640
BWD	*O. longilobus*	Beiwudang, Hebei	114.08	37.06	1160
JNH	*O. longilobus*	Jingnianghu, Hebei	113.98	36.99	650
BXT	*O. longilobus*	Beixiangtang, Hebei	114.22	36.59	520
LFS	*O. longilobus*	Lufengshan, Hebei	113.76	36.49	1020
XTS	*O. longilobus*	Xiantaishan, Henan	113.73	36.18	873
SBY	*O. longilobus*	Shibanyan, Henan	113.72	36.15	720
GJT	*O. longilobus*	Gaojiatai, Henan	113.68	36.11	805
HGS	*O. longilobus*	Honggushan, Henan	113.72	36.00	741
HLT	*O. longilobus*	Heilongtan, Shanxi	113.49	36.01	1054
HDX	*O. longilobus*	Hongdouxia, Shanxi	113.52	35.91	1070
XYG	*O. taihangensis*	Xiyagou, Shanxi	113.60	35.64	1268
DSC	*O. taihangensis*	Dashuangcun, Shanxi	113.44	35.56	868
LQZ	*O. taihangensis*	Linqizhen, Henan	113.94	35.72	873
LSZT	*O. taihangensis*	Linshizuting, Henan	114.01	35.62	530
YMS	*O. taihangensis*	Yunmengshan, Henan	114.09	35.61	1005
GS	*O. taihangensis*	Guanshan, Henan	113.54	35.56	609
BQ	*O. taihangensis*	Baoquanshuiku, Henan	113.49	35.47	895
JYS	*O. taihangensis*	Jingyingsi, Henan	113.20	35.39	1006
QLX	*O. taihangensis*	Qinglongxia, Henan	113.17	35.40	841
SNS	*O. taihangensis*	Shennongshan, Henan	112.82	35.21	1028
WWS	*O. taihangensis*	Wangwushan, Henan	112.27	35.19	3000


## Materials and Methods

### Transcriptome Library Construction

#### Plant Materials

Natural populations of *O. longilobus* (HDX) and *O. taihangensis* (XYG) were collected during July and August in 2015 years across their distribution ranges, respectively (**Figure 1** and **Table 1**). Each individual from the same population was collected from different locations at least 10 m apart. Then mixed leaves of five individuals were collected as a sample. Three samples (biological replicates) were tested for each population, including LR1593, LR1594, and LR1595for *O. taihangensis* and LR1596, LR1597, and LR1598 for *O. longilobus*. And, the fresh and healthy leaves were randomly picked, washed with water and freeze-dried by using liquid nitrogen for RNA extraction.

### RNA Isolation and Quality Verification

Using the RNAout Reagent (CAT#:71203), RNA was isolated from frozen leaves powders and subsequently treated with RNase-free DNase I (Invitrogen). An Agilent 2100 Bioana-lyzer RNA Nanochip with the RNA6000 Nano Lab Chip Kit (Agilent, Santa Clara, CA, United States) was used to verify the quality and quantity of RNA samples. The RNA Integrity Number and the ratio of 28S:18S should reach up to 8.5 and 1.5, respectively. The concentrations and purity of the prepared aqueous RNA were determined by spectrophotometry at λ = 260 and 280 nm, and the criteria was based on OD260/280 between 1.8 and 2.2 and OD260/230 > 1.8.

### Purification of mRNA

Equal quantities of total RNA from the samples were prepared for RNA-Seq. The transcriptome library was constructed using the Illumina TruSeq Stranded mRNA LT Sample Preparation Kit (Illumina, San Diego, CA, United States) according to the manufacturer’s protocol. The total RNA sample was purified by using poly-T oligo-attached magnetic beads to obtain the high quality mRNA. The remaining mRNA was fragmented into small pieces by using divalent cations under elevated temperature in Illumina proprietary fragmentation buffer.

### cDNA Library Construction and Sequencing

First and second strand cDNA were synthesized from poly(A) RNA by using random oligonucleotides and SuperScript II, DNA polymerase I and RNase H, respectively. The remaining 3′ or 5′ overhangs were converted into blunt ends via T4 DNA polymerase and Klenow enzyme. After the adenylation of the 3′-terminal ends of DNA fragments, Illumina PE adapter oligonucleotides were ligated to prepare them for hybridization. Library fragments were purified with AMPure XP system (Beckman Coulter, Beverly, MA, United States) to select cDNA fragments with a length of 200 bp. Using a highly sensitive Illumina PCR Primer Cocktail, DNA fragments ligated adaptor molecules at both ends were selectively enriched in a 10 cycle PCR reaction.

### Library Quantification and Sequencing

Using the high sensitivity Agilent DNA assay on the Agilent Bioanalyzer 2100 system, the products were purified (AMPure XP system) and quantified. According to the vender’s instructions, the clustering of the index-coded samples was performed on a cBot Cluster Generation System by using the Tru PE Cluster Kit V3-cBot-HS (Illumia). After cluster generation, the library preparation was sequenced on a NextSeq 500 High Output Kit (300 cycles).

### *De novo* Assembly, Function Annotation, and Classification

After the removal of raw reads that containing adaptor contamination, low-quality and undetermined bases from each of the datasets (Tong et al., 2013; Xue et al., 2013) and the high-quality clean reads from the samples were *de novo* assembled into contigs and unigenes by using the shout read assembling program called Trinity RNA-Seq Assembler. The GC content analysis was done using in-house perl script (Zhao et al., 2013). The unigenes of the *Opisthopappus* transcriptome were annotated and classified by a sequence similarity search against databases, including the NCBI non-redundant protein database (Nr), Gene Ontology (GO), Cluster of Orthologous Group (COG), Swiss-Prot, and Kyoto Encyclopedia of Genes and Genomes pathway (KEGG) database.

BLASTX tool was used to search and scan the Nr, KEGG, and COG databases for best hits with an *E*-value below the threshold 10-5. Blast2go sofware^1^ was used to analyze the Gene Orthology (GO) and KEGG Orthology (KO) annotations of unigenes. GOSlim terms associated with molecular function, biological process and cellular component of genes were assigned to each assembled transcript.

### Identification of SNP and SSR

Single nucleotide polymorphism with the sequence read coverage of each nucleotide allele were derived from the MIRA contig output file. Only nucleotides with PHRED-scale quality >30 were counted (Ewing et al., 1998). Only DNA sequences positions where the nucleotide allele had a coverage of ≥3 were considered as valid SNPs. The SSRs were screened by using MIcroSAtellite (MISA) identification tools whose minimum number of repeating units was six for di-nucleotides and five for tri-, tetra-, penta- and hexa-nucleotides. The mono-nucleotide repeats were generally not very informative and thus were not considered for the analysis.

### Development of SNP Markers

#### Plant Materials

Six natural populations each of *O. longilobus* (XT, JNH, BXT, LFS, SBY, and HDX) and *O. taihangensis* (XYG, BQ, QLX, SNS, WWS, and LQZ) were collected on July and August in 2015 years across their distribution ranges, respectively (**Figure 1** and **Table 1**). Each individual from the same population was collected from different locations at least 10 m apart. For each population, fresh and healthy leaves were randomly picked from 15 to 20 individuals, and then stored at -20°C until DNA extraction.

### DNA Extraction

Total genomic DNA was extracted from young leaves by using a modified CTAB procedure (Doyle, 1987; Xu et al., 2006). The DNA quality of samples was determined by electrophoresis on 0.8% agarose gel according to Sambrook and Russell (2001). The extracted DNA samples were diluted to 30 ng/μL and stored at -20°C until use.

### Primer Design and Screening, SNP Amplification and Sequencing

On the basis of the transcriptome sequence results of the predicted SNP loci, SNP primers were designed by Primer Premier5 software. The primers were synthesized by Sangon Biotech (Shanghai, China) and tested using nine individuals, which were randomly selected from three populations of *Opisthopappus* Shih located in geographically distinct locations. The PCR-product was resolved on 2% agarose gel after the PCR reaction. The product with a single and bright band was used for unidirectional sequencing. Finally, the effective primers for subsequent analysis were listed in **Supplementary Table S1**.

The 20 μL PCR reaction system contained 2 μL of 30 ng/μL DNA, 10 μL of 2 × Taq PCR MasterMix, 1.0 μL each of 10 μmol/L primer, and 6.0 μL ddH_2_O. PCR was carried out as follows: 94°C for 5 min, 94°C for 1 min, 72°C for 1 min and 72°C for 1.5 min. The cycle was repeated for 35 times and with a cycle of extension at 72°C for 10 min.

### Data Analysis

The DNA sequences were aligned using Clustal X 1.81 and Bio Edit v7.0.9 and then adjusted manually (Thompson et al., 1997; Hall, 1999). The nucleotide diversity (π) and haplotype diversity (*H*d) were calculated for each population by using the DnaSP software (Rozas et al., 2003). The parameters of population diversity, gene diversity (*H*_S_), and total gene diversity (*H*_T_) were estimated by following the methods described by Pons and Petit (1996) by using the program PERMUT^2^. The pairwise *F*_ST_ values were obtained from the AMOVA analysis by using the ARLEQUIN software (Excoffier et al., 2005), with the significance tested by 1,000 permutations. To further examine the genetic relationships among populations, a dendrogram was constructed based on the genetic distance matrix by using the unweighted pair-group method with arithmetic mean (UPGMA) clustering algorithm. Bootstrap analysis of UPGMA tree was performed using MEGA5 with 1,000 replicates (Tamura et al., 2007). The population structure was analyzed using the software package STRUCTURE v2.4 (Budak et al., 2004). The calculation was carried out under the linkage and uncorrelated allele frequency model. A burn-in period of 10,000 generations, followed by 50,000 iterations, was used to cluster the population. The assumed number of populations (*K*) was set from 2 to 10.

## Results

### Sequencing and Reads Assembly

In this study, the transcriptomes of *O. taihangensis* tissues were analyzed using the Illumina HiSeq 2000 platform with 150 bp paired-end reads. Approximately 34.9 million clean reads filtered were obtained from raw reads by removal of adaptors, generating more than 5.23 giga-bases (Gb) of sequences. The statistics of raw reads are shown in **Table 2**. High-quality sequencing data was reflected by the average Q20 of 97.85%, while Q30 was more than 95%. Moreover, the percentage of unknown nucleotide (*N* percentage) was about 0.01% (**Table 2**).

**Table 2:** Overview of output statistics (I) on *Opisthopappus* Shih transcriptome sequencing.

Sample		Total raw reads	Total clean reads	Q20 percentage	Q30 percentage	*N* percentage
*O. taihangensis*	LR1593	40019204	39768606	97.74	95.08	0.014699
	LR1594	34846310	34664504	97.87	95.34	0.014688
	LR1595	30400160	30235546	97.93	95.40	0.014394
*O. longilobus*	LR1596	32754732	32564942	97.83	95.22	0.014546
	LR1597	35364340	35201284	97.99	95.53	0.014401
	LR1598	29932178	29758656	97.88	95.32	0.014462
Mean		33886154	33698923	97.87	95.32	0.014532


For *O. longilobus*, more than 32.5 million clean 150 bp paired-end reads were produced and a total of 4.88 Gb of sequences was generated. High quality sequencing data was reflected by the average Q20 of 97.90% and the percentage of unknown nucleotide (*N* percentage) was about 0.05% (**Table 2**).

An average of 33,698,923 clean reads per library was obtained from 33,886,154 raw reads reaching the average Q20 of 97.87%, which were clustered and *de novo* assembled into 98,12,450 contigs with size range of 78–10,531 bp and average N50 of 488 bp (**Table 2**). Using the Trinity method, a non-redundant set of 800,793 transcripts (unigenes) with an average length of 445 bp was generated from high-quality trimmed reads, among which N50 and N90 were 488 and 241 bp in length, respectively (**Table 3**). The lengths of the 71,566 transcripts (62.65%) were between 200 and 500 bp and 7,375 (6.46%) transcripts were longer than 2 kb (**Supplementary Figure S1**).

**Table 3:** Overview of output statistics (I) on *Opisthopappus* Shih transcriptome sequencing.

	Contig	Transcript	Unigene
Total length (bp)	782,268,047	356,394,553	27,229,229
Sequence number	9,812,450	800,793	33,975
Max. length (bp)	10,531	10,531	9,255
Mean length (bp)	79.72199063	445.0520334	801.4725673
N50 (bp)	112	488	1,140
N50 sequence no.	1,369,927	197,189	7,746
N90 (bp)	38	241	363
N90 sequence no.	7,355,672	642,185	24,072
GC%	50.68	51.29	47.19


### Classification of Known and Novel Protein-Coding Genes

After filtering the reads with Bowtie 2, the number of reads mapped to the orthologous region of each gene in proper pairs exceeded half the total reads and reached 45,161,366 (71.54%) and 47,663,788 (69.42%) for *O. taihangensis* and *O. longilobus*, respectively, which indicate that most of the reads obtained were available to functional and expression pattern analysis.

Based on the clustering pattern and the library specificity, 33,974 standard unigenes were identified with a mean size of 801 bp and N50 of 1140 bp (**Table 3**). The overall length distribution of assembled unigenes are shown in **Supplementary Figure S2**. All unigenes were subjected to BLAST searches against five public databases (NR, NCBI non-redundant protein sequences; Go, Gene Ontology; KEEG, Kyoto Encyclopedia of Genes and Genome; eggNOG, evolutionary genealogy of genes: Non-supervised Orthologous Groups; Swiss-Prot) for functional annotation, prediction and classification. A total of 33,974 unigenes were annotated in NR and GO database, and 4,857 (14.3%), 10,357 (30.48%), and 30,789 (90.62%) of them showed homologous matches against the KO, eggNOG and Swiss-Prot database, respectively. Meanwhile, 11,306 unigenes (33.28%) were annotated in at least one database. Only about 990 of the unigenes (2.91%) were simultaneously annotated in all databases, which could mainly be attributed to the existence of large number of sequences with short lengths of 100–500 bp, resulting in limitedly and falsely annotated protein-coding genes, or lack of adequate genome information about the genomes and transcriptomes of *Opisthopappus* in the public databases (**Supplementary Table S2**).

In addition, 24,500 unigenes with lengths longer than 300 bp were BLAST-annotated, whose sequence can be used as the training set for discovery of novel protein coding genes. For example, based on eggNOG, a total 23,400 unigenes had no matches after BLAST search, which were predicted to contain coding sequence longer than 300 bp and thus classified as novel protein coding genes. The remaining unigenes were expected to consist of either messenger like non-coding RNA or fragments of untranslated region of protein coding genes (**Supplementary Table S2**).

### Identification of Known Protein Coding Genes

**FIGURE 2:**
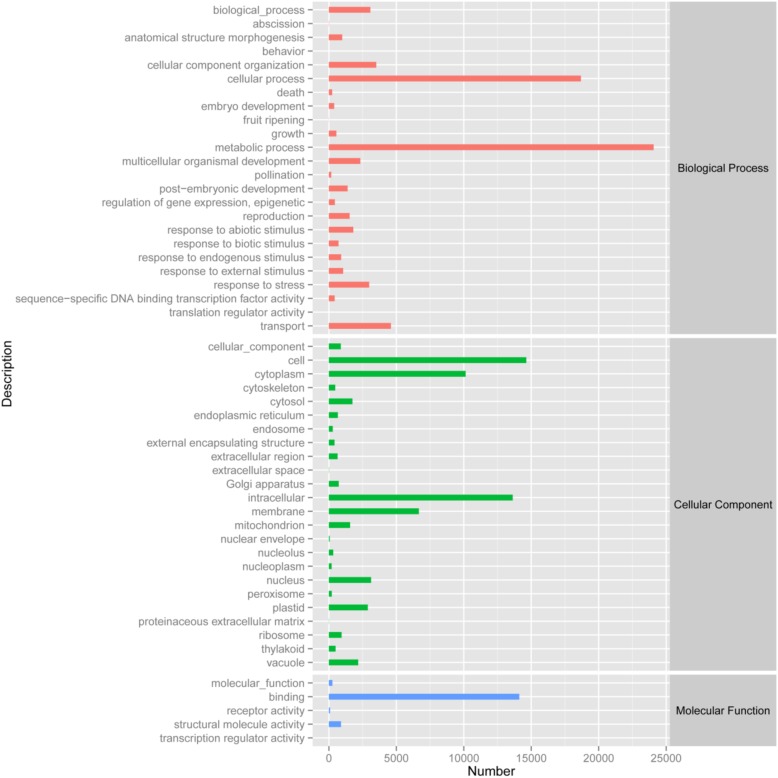
Gene Ontology classification of the 33,975 protein annotated unigenes. Unigene sequences were systematically classified into GO sub-categories under the biological process, cellular component and molecular function GO catalog system. Each bar represents the relative abundance of unigenes classified under each sub-category.

By analyzing the annotation and characteristics of blast homology search of unigenes against the GO database, the results showed that 33,974 unigenes (Biological process: 5,231 unigenes; Cellular component: 4,689 unigenes; Molecular function: 6,198 unigenes) could be assigned to 53 GO groups and the function classification of unigenes across all groups was presented in **Figure 2**. A highly represented biological process ontology included biological process, cellular component organization, metabolic process, response to stress and transport, whose ontology terms were represented by more than 2,500 unigenes. The unigenes assigned cellular component category was mainly compartmentalized in the cell, cytoplasm, intracellular and membrane. Additionally, GO analysis of molecular function showed several significantly matched unigenes to binding function (**Figure 2**).

Within eggNOG annotation, ontology represented by more than 900 unigenes were mainly distributed in the clusters of translation, ribosomal structure and biogenesis; post-translational modification, protein turnover, and chaperones; general function prediction only and signal transduction mechanisms (**Supplementary Figure S3**).

A KEGG functional enrichment analysis was performed and about 4,875 unigenes were annotated in 428 pathways. The activated and enriched pathways showed that “infectious diseases” (605 unigenes), “carbohydrate metabolism” (588 unigenes), “translation” (527 unigenes) and “signal transduction” (541 unigenes) exhibited the higher levels in metabolism, genetic information processing, environmental information processing and human diseases pathways. Besides, nearly 400 unigenes primarily involved in “cancers,” “folding, sorting, and degradation,” and “energy metabolism” were enriched in related metabolic pathway, just behind “cell growth and death” process, “lipid metabolism,” “replication and repair,” and “transport and catabolism” (**Supplementary Figure S4**).

### Analysis of Differentially Expressed Genes

**FIGURE 3:**
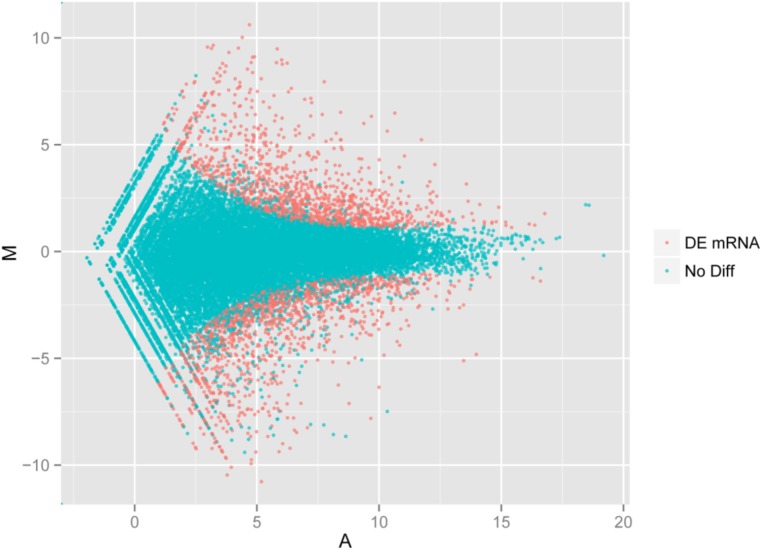
Digital gene expression of the two species. The *x* and *y*-axis are the log10 of the normalized expression level (RPKM) of the unigene in the indicated tissue. Each point represents a unigene. Red points indicate the significant expressed unigenes with the absolute value of log2 ≥ 1 and FDR ≤ 0.001. Blue points indicate insignificant differentially expressed unigenes.

**FIGURE 4:**
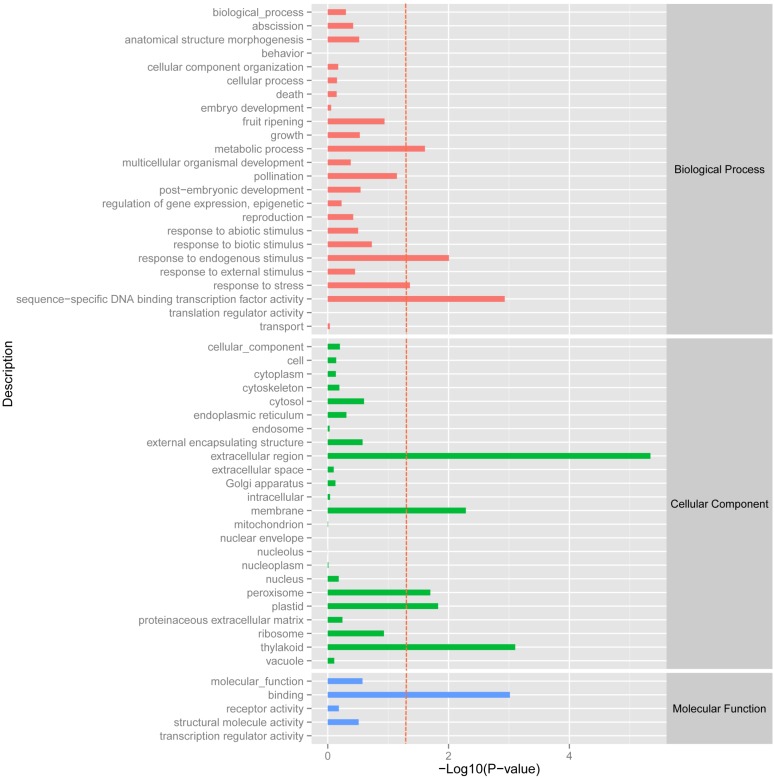
GOSlim enrichment analysis of different expression unigenes. Different expression unigenes were systematically classified into GO sub-categories under the biological process, cellular component and molecular function GO catalog system. The *x*-axis is the log10 of the normalized expression level (RPKM) of unigene in the indicated tissue. The red dotted line in this figure indicates that the *p*-value was 0.05, and *p*-values below 0.05 are regarded as significant. Each color represents the different GO sub-categories.

**FIGURE 5:**
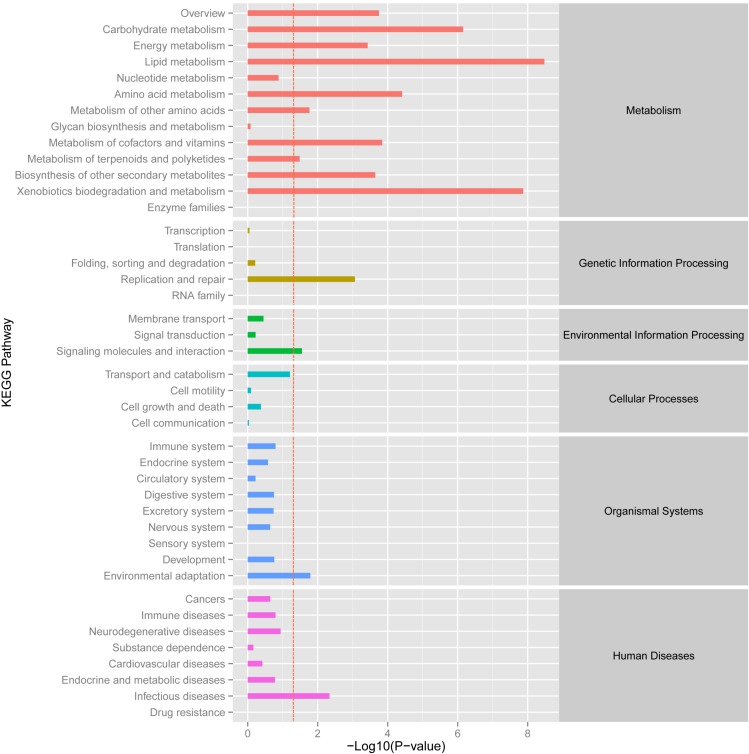
KEGG enrichment analysis of different expression unigene. Different expression unigenes were systematically classified into sub-classes of KEGG pathway. The *x*-axis is the log10 of the normalized expression level (RPKM) of unigene in the indicated tissue. The red dotted line in this figure indicated that the *p*-value was 0.05, and *p*-values below 0.05 are regarded as significant. Each color represents the different sub-classes of KEGG pathway.

Using the DESeq package to explore the similarities and differences, a total of 3,410 differentially expressed transcripts were detected, including 1,925 up-regulated and 1,485 down-regulated genes (**Supplementary Table S3**). Moreover with the ggplot2 of the R software package, the result was shown by a direct MA plot (**Figure 3**). The corresponding differentially expressed genes were annotated and analyzed in GO and KEGG database. The results showed a significantly difference in the expression of extracellular region primarily associated with the cellular component. And the variation of the thylakoid, binding and sequence-specific DNA binding transcription factor activity were similar to each other and were significantly enriched in cellular component, molecular function and biological process (**Figure 4**). Besides, the largest number of unigenes involved in the majority of the metabolic pathway, including lipid metabolism, xenobiotics biodegradation and metabolism and carbohydrate metabolism and so on, exhibited significant change in the expression of related genes. However, in the other pathway, the results showed that unigenes only associated with replication and repair, signaling molecules and interaction, environmental adaptation and infectious diseases processes had a higher activity (**Figure 5**).

**FIGURE 6:**
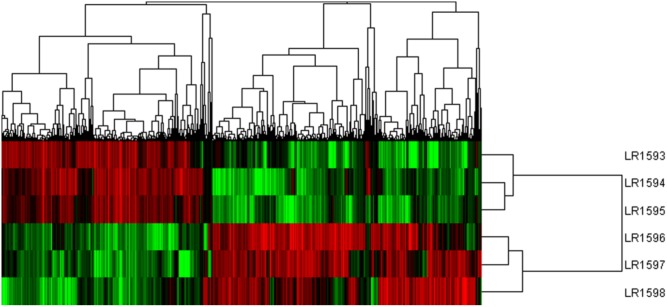
The cluster analysis of different gene expression of the three samples of *O. longilobus* and *O. taihangensis*. Hierarchical cluster analysis of gene expression based on the log ratio RPKM data. The hierarchical clustering method based on pairwise average-linkage analysis. Up-regulation and down-regulation of genes are indicated in red and green color. LR1593-LR1595 samples are *O. longilobus*, and LR1596-LR1598 samples are *O. taihangensis*.

Considering the different gene expression patterns measured by systematic cluster analysis, the results of hierarchical clustering indicated that the gene expression profiles could be divided into two distinct groups. LR1594 and LR1595 were clustered into one group, then LR1593 was to join in the first group. Additionally, LR1596 and LR1597 showed a high similarity, classified into the same cluster and were then gathered into another class with LR1598 (**Figure 6**).

### Identification of SNP and SSR Markers

The incidences of substitution in SNPs are generally higher than those of transversion. However, the estimation of the number of putative SNPs of *Opisthopappus* Shih was 71,804, and the transition-to-transversion (t/v) ratio was estimated to be 0.22. The results showed that transitions were not expected to be more frequent than transversions (Gojobori et al., 1982; Bainbridge et al., 2011; Ophir et al., 2014). This observation might underlie the fact that the predicted SNP sites were obtained mostly from coding sequences, where the natural selection on transition substitutions were higher than that on transversion substitutions.

Additionally, a total of 1,444 SSRs were identified in 800,793 transcripts of *Opisthopappus* Shih. In SSRs, tri-nucleotide repeats (37.52%, 992) were the most abundant motifs, followed by di-nucleotide repeats with 15.51% (410). ACC/GGT (20.9%), ATC/ATG (18.6%), AAG/CTT (12.2%), and AAC/CTT (11.1%) were the main types of motif in tri-nucleotide repeats. The di-nucleotide repeat motifs of AT/AT were the predominant repeat types (65.4%), followed by AG/CT (22.9%) and CG/CG was the least abundant (0.98%).

### Population Genetic Analysis of *Opisthopappus* Shih by Using SNP Markers

Genetic variance analysis using the SNP markers showed that a low level of gene flow between *O. longilobus* and *O. taihangensis* (*N*m = 0.07). A high genetic diversity in *Opisthopappus* Shih populations was detected. The parameters *H*_T_, *V*_T_, and *V*_S_ were 0.989, 1.025, and 0.368, respectively. The estimated haplotype diversity (*H*_d_) and nucleotide diversity (π) was 0.984 and 0.00737, respectively (**Table 4**). Meanwhile, the genetic characteristic values of *O. longilobus* were higher than those of *O. taihangensis*. Among populations of *O. longilobus*, the nucleotide diversity (π) ranged from 0.00146 to 0.00344, and the JNH population had the highest π value. The nucleotide diversity (π) was estimated within the *O. taihangensis* as a whole (0.00149) and within populations, ranging from 0 to 0.00172, and the SNS population had the highest π value (**Table 4**).

**Table 4:** The estimated diversity indexes of *Opisthopappus* Shih populations based on SNP data.

Populations	Sample size	Haplotype diversity (*H*_d_)	Nucleotide diversity (π)
HDX	20	1	0.00198
BXT	16	1	0.00271
JNH	15	1	0.00344
LFS	17	1	0.00271
XT	16	1	0.00146
SBY	15	1	0.00328
*O. longilobus*		0.99080	0.00318
WWS	16	1	0
LQZ	15	0.9	0.00167
XYG	18	1	0.00094
SNS	15	0.9	0.00172
BQ	17	0.9	0.00115
QLX	15	0.7	0.00042
*O. taihangensis*		0.94071	0.00149
*Opisthopappus* Shih		0.98434	0.00737


In addition, a significantly high level of genetic differentiation was detected between the populations of *O. longilobus* and *O. taihangensis* (average pairwise *F*_ST_ = 0.769). The genetic differentiation between the XT population of *O. longilobus* and WWS, XYG and QLX populations of *O. taihangensis* were up to more than 0.9, whereas a relatively low genetic differentiation was observed within *O. longilobus* and *O. taihangensis* populations (**Supplementary Table S4**).

**FIGURE 7:**
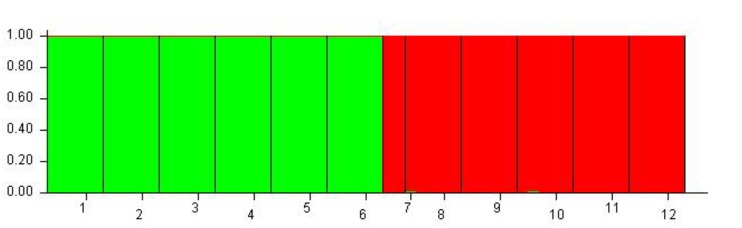
Estimated genetic structure for *K* = 2 obtained with the STRUCTURE program for 12 populations of *Opisthopapus* Shih based on SNP. Each vertical bar represents a single individual, whereas colored areas correspond to distinct genetic cluster. The number represents the populations of *Opisthopappus* Shih, including 1. HDX 2. BXT 3. JNH 4. LFS 5. XT 6. SBY 7. WWS 8. LQZ 9. XYG 10. SNS 11. BQ 12. QLX.

**FIGURE 8:**
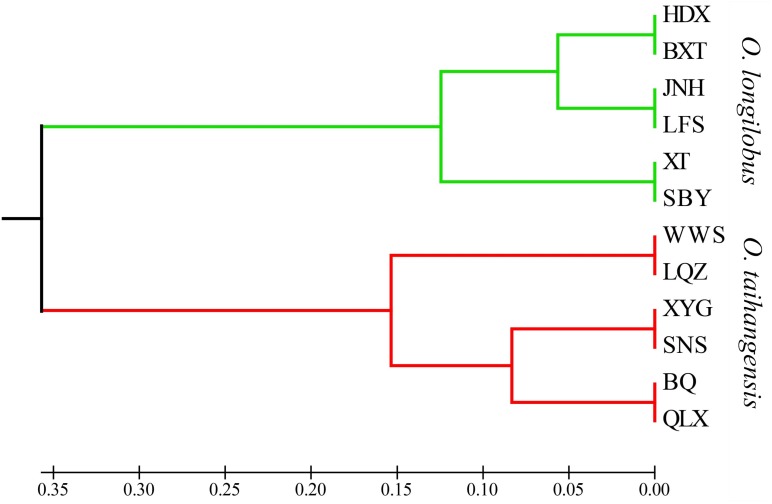
Phylogenetic dendrogram based on genetic distance by the UPGMA cluster in the 12 populations of *Opisthopappus* Shih.

Although 12 populations were initially analyzed, the Bayesian analysis allowed the identification of only two distinct genetic groups, when the highest *ΔK* value was achieved with *K* = 2 (**Supplementary Figure S5**). The six populations of *O. longilobus* were grouped in the first cluster (green, **Figure 7**), and six populations of *O. taihangensis* were grouped in the second cluster (red, **Figure 7**), indicating a strong genetic structure. Similarly, as shown in **Figure 8**, the UPGMA cluster analysis assigned the 12 populations into two groups, which clearly separated the *O. taihangensis* populations from the *O. longilobus* populations in term of genetic distance.

## Discussion

### Annotation and Classification of Transcriptomic Data

Owing to the increasing speed, decreasing price, and improving efficiency of DNA sequencing technologies, the “explosion” of next-generation sequencing data can facilitate a faster accumulation of raw DNA sequence data than subsequent time-consuming genomic analysis (Andrews and Luikart, 2014). Moreover, the annotated libraries were continually established and enriched, which would make the raw sequence available to a broader scientific community. In this study, a *de novo* assembly and annotation of *Opisthopappus* Shih transcriptome by using Illumina platform allowed us to analyze and identify the transcripts expressed during population differentiation. For comprehensive annotation, a similarity search against various known databases was subjected to *Opisthopappus* Shih transcriptome.

The functions of transcripts in *Opisthopappus* Shih were successfully annotated by NR and GO analyses, and 33,974 unigenes were assigned to 24 GO categories including three main divisions (biological process, cellular component, and molecular function). Among the biological processes, “metabolic process” (24,073 unigenes) has the largest portion, followed by “cellular process” (18,683 unigenes) and “transport” (4,597 unigenes). A total of 3,508 unigenes and 3,075 unigenes were involved with “cellular component organization” and “biological process,” respectively (**Figure 2**). These results might reflect the actual biological characteristics and differentiation of *O. taihangensis* and *O. longilobus*. In addition, 2,984 unigenes related to biological process were associated with “response to stress,” which could suggest the unique environmental adaptability for these two species, which should be explored as candidates for studying their role in response to particular stress or trait. According to the cellular component category, a high proportion of the transcripts were assigned to “cell” (14,636 unigenes), and a significant proportion of the transcripts were connected with “intracellular” (13,629 unigenes), hence, different and special physiological process might be related to the corresponding biological process in the two species. Besides, in the molecular function category, “binding” (14,115 unigenes) occupied the largest percentage, and might play an important role in regulating the important physiological functions of *O. taihangensis* and *O. longilobus* (**Figure 2**).

More than 30.48% of transcripts were classified into 24 functional categories based on COG of proteins. “Post-translation modification, protein turnover and chaperones” and “translation, ribosomal structure and biogenesis” were remarkably and similarly represented and the proportion of these categories in our study was quite higher than other relative species, which meant that basic physiological and metabolic process played a very important and extremely active role in *Opisthopappus* Shih (**Supplementary Figure S3**) (Lai and Lin, 2013; Wang et al., 2013; Wei et al., 2014).

In addition, KEGG analysis predicted that the 4,857 expressed genes were mapped onto 428 pathways, and the most enriched sequence was “metabolic pathways” (**Supplementary Figure S4**). A total of 588 unigenes primarily involved in carbohydrate metabolism could provide energy and material for organisms, such as, starch and sucrose metabolism, amino-sugar and nucleotide sugar metabolism and glycolysis/gluconeogenesis and so on, which revealed that the above signaling pathways were significantly important to the development and growth process of *O. taihangensis* and *O. longilobus*. Furthermore, with “amino acid” as a fundamental nutrient for protein synthesis and cell growth, about 400 unigenes involved in related metabolism and the “translation” pathway (527 unigenes) were also highly expressed, which suggested that both pathways were complementary and would coordinately regulate cell/organism growth and development. In addition, “signal transduction” (541 unigenes) was also enriched in “environmental information processing” pathway, and it showed that *Opisthopappus* Shih was likely to receive a chemical or physical signal and transmit through a cell as a series of molecular events in response to complicated environmental changes and barren habitat. Besides, the higher levels and activity of pathways were also exhibited in “energy metabolism” (313 unigenes), “lipid metabolism” (271 unigenes), “folding, sorting and degradation” (361 unigenes), “replication and repair” (254 unigenes), “transport and catabolism” (241 unigenes) and “cell growth and death” (285 unigenes) process. These results provided support that the development and growth of *O. taihangensis* and *O. longilobus*, like that of other species involved a highly sophisticated system of tissues and cells, which required the cooperation of several metabolic pathways and established relationship between the structure, function and regulation in complex cellular networks (**Supplementary Figure S4**).

### Comparative Transcriptomic Analysis

The newly developed approaches of deep sequencing-based plant transcriptome have significant advantages for investigating and revealing differential gene expression. In order to obtain more accurate and more reliable experimental data by RNA-Sequencing, impact of geographical environment interference on expression of exogenous genes needs to be seriously considered. Generally, comparison with wild populations, growth environment conditions of domesticated and cultivated species can be controlled and intervened artificially according to specific experimental requirements. *Opisthopappus* Shih, an endangered cliff genus endemic to the Taihang Mountains of China, grows in the crevice of cliffs and is in fragmented distribution. Therefore, in this study, XYG population of *O. taihangensis* and HDX population of *O. longilobus* locating in region with geographical proximity, similar elevation and homogeneous environment, were carefully chose as sampling sites for comparative transcriptome analysis, which efficiently decrease disturbances from circumstance factors (**Figure 1** and **Table 1**).

We have identified a set of 3,410 genes, which were differentially expressed in the biological, cellular and molecular processes of *O. taihangensis* and *O. longilobus*, including 1,925 up-regulated genes and 1,485 down-regulated genes (**Supplementary Table S3**). The significant difference in the physiological activity of the extracellular region detected in *Opisthopappus* Shih suggested that related physiological and biological processes might be remarkably associated with the response to external stimulus, which contributed to the screening of essential candidate genes for improving tolerance in cultivars (Hu, 2008) (**Figure 4**). *O. taihangensis* and *O. longilobus* as endemic species of China, have a stronger adaptation capability and higher tolerance, especially to drought, barren soil, freezing, and plant diseases (Wang et al., 1978). Therefore, further studies are needed to confirm the potential molecular mechanisms associated with candidate genes and to uncover the exact functions of relevant signaling pathways in *Opisthopappus* Shih.

The KEGG pathway enrichment analysis of differentially expressed genes showed that genes were mainly involved in the lipid metabolic pathway and associated with replication, repair and environmental adaptation (**Figure 5**). For example, in the lipid metabolic pathway, 21 and 39 genes were up-regulated genes in *O. taihangensis* and *O. longilobus*, respectively. The only upregulating cytochrome *P450* and *CYP710A* in *O. longilobus* encode sterol C-22 desaturase and control the sterol and steroid biosynthesis, which are isoprenoid-derived lipids and are produced via the mevalonate pathway (Morikawa et al., 2006). Their whole body contains aromatic oil, which makes *Opisthopappus* Shih important medicinal and ornamental plants. Therefore, the validation of *P450* and *CYP710A* gene function in *Opisthopappus* Shih can reveal the physiological difference between *O. taihangensis* and *O. longilobus*. Similar results were confirmed in xenobiotics biodegradation and metabolism. The *GST* gene (glutathione S-transferase), *adh* gene (alcohol dehydrogenase) and *adhC* gene [S-(hydroxymethyl) glutathione dehydrogenase] were involved in the metabolism of xenobiotics through the cytochrome P450 pathway for natural and xenobiotic substrates stress response (Schalk et al., 1997). However, *GST* and *adh* were only up-regulated in *O. longilobus*, whereas *adhC* was only up-regulated in *O. taihangensis*, which indicated the difference in the molecular mechanism in xenobiotics biodegradation pathway.

HSP90A (Heat shock protein 90) is a multi-functional molecular chaperone, that plays an essential role in both cellular metabolism and various stress response, which up-regulates only in *O. taihangensis* (Ye and Qiang-Hua, 2012) (**Figure 5**). *Opisthopappus* Shih naturally grows on cliff cracks, rock gaps in open forests below cliffs and infertile soil, which possesses protective effect against drought and leanness. It speculated that HSP90A protein might be involve in environmental adaptation physiological process in *Opisthopappus* Shih. Focusing on the resistance and tolerance of HSP90A protein, *O. taihangensis* and *O. longilobus* should be able to adapt to their surroundings and present the environmental adaptation through different molecular mechanism.

The gene expression profiles between the population of *O. taihangensis* and *O. longilobus* were divided into two distinct groups (**Figure 6**). *Opisthopappus* Shih was mainly distributed in Taihang Mountains, which was affected by the uplift of Qinghai-Tibet plateau and has experienced the quick rising during glacial period. During the long-term evolution of *Opisthopappus* Shih, dramatic geological changes and significant topographical barriers could limit the large-scale population expansion and gradually introduce heterogeneity within different populations of *O. taihangensis* and *O. longilobus*. These two species, as herbaceous plant, should be highly sensitive to changes and inevitably induce high expression level of a subset of environmental adaptation pathway-related genes.

Therefore, according to these results, activity differences exist in many pathways between *O. taihangensis* and *O. longilobus*. This phenomenon suggests that the diversification of basic physiological and biochemical metabolism occurs between them, which is beneficial to further explore the difference in the characteristics of the molecular mechanism between *O. taihangensis* and *O. longilobus*.

### Development and Validation of Molecular Markers

Population genetic analysis in *Opisthopappus* Shih have been carried out by using various molecular markers, such as RFLP, RAPD, ISSR, cpSSR, and SRAP (Guo et al., 2013; Wang, 2013; Wang and Yan, 2013). However, no definitive conclusions were established to illustrate interspecific differences and confirm the taxonomy status between *O. longilobus* and *O. taihangensis*.

Compared with AFLP, RFLP, and SSR markers, SNPs are co-dominant markers, that are widely distributed in the genome, and the probability for each nucleotide mutation is about 10^-9^ (Mooney, 2005). Due to their abundance across the whole genome and their potential for cost-effective high-throughput genotyping, SNPs are also increasingly used to study the history of a population and the evolution of species (Pavy et al., 2013). The *Opisthopappus* Shih transcriptome exhibits an increased depth of coverage and high-confidence nucleotide calls and facilitated rapid discovery of informative loci, a large number of SNPs (71,804) have been predicted in this study.

At present, we have developed eight effective SNP markers for population genetic analysis of *Opisthopappus* Shih based on the transcriptome data (**Supplementary Table S1**). The higher levels of genetic diversity in *Opisthopappus* Shih were observed. Considering *O. longilobus* and *O. taihangensis* both grow on the cracks of the steep cliffs, the strict ecological surrounding and diverse habitation contribute to the high genetic variation. Moreover, there have a common patchy distribution between the two species of *Opisthopappus* Shih. *O. longilobus* is mainly distributed in Hebei Province, the south of the Taihang Mountains belonging to the warm temperate zone with semi-humid continental monsoon climate, whereas *O. taihangensis* is distributed in Shanxi and Henan Province, the north of the Taihang Mountains belonging to the temperate continental monsoon climate (Wang et al., 1978; Ding and Wang, 1997). Thus, the isolated populations may be more capable of maintaining genetic variation to adapt to ecological characteristics (Chen et al., 2004; Hilfiker et al., 2004).

In addition, we found that there existed a low level of gene flow and the significantly high level of genetic differentiation between the populations of *O. longilobus* and *O. taihangensis*, which were clearly grouped into two distinct genetic clusters. And the higher divergence between *O. longilobus* and *O. taihangensis* was possibly related to the early split of the two major lineages, as indicated by UPGMA. Because of harsh climate changes and the tectonic events of the Taihang Mountain during the Miocene, both genetic drift and inbreeding may occur with drastically reduced population sizes of *O. longilobus* and *O. taihangensis* (Hu, 2008). And *O. taihangensis* and *O. longilobus* might occupy different niches due to difference in growth habits, the uplift and transition of paleo-vegetation of Taihang Mountains as a genetic barrier might restrict the gene flow in fragmented populations and maintain significant population differentiation (Wang and Yan, 2014).

Comparative efficiency of SRAP, cpSSR, ITS, and SNP markers for population differentiation of *Opisthopappus* Shih, we found that the *O. taihangensis* populations could be clearly separated from the *O. longilobus* populations by using SNP markers (Murray and Thompson, 1980; Thomas and Kejariwal, 2004; Han, 2013). The result was consistent with the record of Flora Reipublicae Popularis Sinicae (FRPS) and supported the conclusion that *Opisthopappus* Shih included two species: *O. longilobus* and *O. taihangensis*. However, contrary to the taxonomic status in the Flora of China, *O. longilobus* and *O. taihangensis* were merged into one species. Therefore, more in-depth studies are still needed to provide more definitive evidence, which would deeply reveal the evolutionary relationships of species within *Opisthopappus* Shih and accurately validate the record in FRPS. For example, screening and development of SSRs markers based on transcriptome sequences contribute to the large-scale spatial genetic structure studies. At present, a total of 2,644 SSRs variants are obtained, and a total of 1,444 SSRs with a minimum length of 2–5 nucleotides are predicted in *Opisthopappus* Shih transcripts, of which the most abundant are tri-nucleotide and dinucleotides repeats. Thus, the availability of the whole transcriptome sequence for identifying of SSRs holds an immense potential for phylogeographical analysis in *Opisthopappus* Shih.

## Conclusion

In conclusion, some studies believe that the origin of the mountain flora could be traced back to a high antiquity, and what is more, the flora and vegetation are remarkably evolved and derivative to a certain significant extent. *O. taihangensis* and *O. longilobus* experienced selective pressures under heterogeneous environment, which could be appropriate as an important model for mechanistic studies of a diverse array of ecological and evolutionary questions. In this study, the differential morphology, physiology and metabolic activity and expressed genes, high-quality SNP and SSR library made publicly available will not only contribute to identify important functional genes based on genetic differentiation and variation, allele frequencies indicative of selective sweeps, or linkage disequilibrium in long haplotype blocks, but will also facilitate in revealing a history of population expansion and trait evolution of *Opisthopappus* Shih. Therefore, using sequencing technology, this transcriptome assembly data would promote a substantial increase on the extensive genetic information and functional genomic resources available to *Opisthopappus* Shih. The *Opisthopappus* Shih transcriptome will be useful in future studies on local adaptation, speciation, genome evolution, quantitative trait variation, and investigation of the genetic basis of phenotypic traits.

## Author Contributions

MC, JH, and SW performed the experiments. WC, ZF, and JL collected and analyzed the transcriptome data. MC, JH, and WC wrote the manuscript. MC, JH, and YW edited the manuscript.

## Conflict of Interest Statement

The authors declare that the research was conducted in the absence of any commercial or financial relationships that could be construed as a potential conflict of interest.

## Footnotes

1 https://www.blast2go.com/

2 https://www.winsite.com/Home-Education/Science/PerMut-Software/

## References

[B1] Andrews K. R. Luikart G. (2014). Recent novel approaches for population genomics data analysis. *Mol. Ecol.* 23 1661–1667. 10.1111/mec.12686 24495199

[B2] Bainbridge M. N. Wang M. Wu Y. Newsham I. Muzny D. M. Jefferies J. L. (2011). Targeted enrichment beyond the consensus coding DNA sequence exome reveals exons with higher variant densities. *Genome Biol.* 12:R68. 10.1186/gb-2011-12-7-r68 21787409 PMC3218830

[B3] Barker M. S. Vogel H. Schranz M. E. (2009). Paleopolyploidy in the Brassicales: analyses of the cleome transcriptome elucidate the history of genome duplications in *Arabidopsis* and other Brassicales. *Genome Biol. Evol.* 1 391–396. 10.1093/gbe/evp040 20333207 PMC2817432

[B4] Budak H. Shearman R. C. Parmaksiz I. (2004). Molecular characterization of Buffalo grass germplasm using sequence-related amplified polymorphism markers. *Theor. Appl. Genet.* 108 328–334. 10.1007/s00122-003-1428-4 13679978

[B5] Chen X. D. Li Y. D. Luo T. S. Lin M. X. Sun Y. X. (2004). Study on biomass and net primary productivity of *Podocarpus imbricatus* plantation in Jianfengling. *For. Res.* 17 598–604.

[B6] Ding B. Z. Wang S. Y. (1997). *Flora of Henan, Zhengzhou.* Beijing: Henan Science and Technology Press.

[B7] Doyle J. J. (1987). A rapid DNA isolation procedure for small quantities of fresh leaf material. *Phytochem. Bull.* 19 11–15.

[B8] Ewing B. Hillier L. Wendl M. C. Green P. (1998). Base-calling of automated sequencer traces using phred: I. Accuracy assessment. *Genome Res.* 8 175–185. 10.1101/gr.8.3.175 9521921

[B9] Excoffier L. Laval G. Schneider S. (2005). Arlequin version 3.0: an integrated software package for population genetics data analysis. *Evol. Bioinform.* 1 47–50. 10.1177/117693430500100003 19325852 PMC2658868

[B10] Gao Y. H. Dai P. F. Ji Z. F. Han X. Wang Y. L. (2011). Studies on pollen morphology of *Opisthopappus shih*. *Acta Bot. Boreali Occidentalia Sin.* 31 2464–2472.

[B11] Gojobori T. Li W. H. Graur D. (1982). Patterns of nucleotide substitution in pseudogenes and functional genes. *J. Mol. Evol.* 18 360–369. 10.1007/BF01733904 7120431

[B12] Guo R. M. Zhou L. H. Zhao H. B. Chen F. (2013). High genetic diversity and insignificant interspecific differentiation in *Opisthopappus shih*, an endangered cliff genus endemic to the Taihang Mountains of China. *Sci. World J.* 2013:275753. 10.1155/2013/275753 24453824 PMC3876899

[B13] Hall T. A. (1999). BioEdit: a user-friendly biological sequence alignment editor and analysis program for Windows 95/98/NT. *Nucleic Acids Symp. Ser.* 41 95–98.

[B14] Han X. (2013). *Study on Genealogical Geography of Endangered Opisthopapus shih.* Master thesis, Shanxi Normal University, Xi An.

[B15] Hilfiker K. Gugerli F. Schutz J. Rotach P. Holderegger R. (2004). Low RAPD variation and female-biased sex ratio indicate genetic drift in small populations of the dioecious conifer Taxus baccata in Switzerland. *Conserv. Genet.* 5 357–365. 10.1023/B:COGE.0000031144.95293.1b

[B16] Hu X. (2008). *Preliminary Studies on Inter-Generic Hybridization within Chrysanthemum in Broad Sense (III).* Master thesis, Forestry University, Beijing.

[B17] Jia R. Z. Wang Y. L. (2015). Leaves micromorphological characteristics of *Opisthopappus taihangensis* and *Opisthopappus longilobus* from Taihang Mountain, China. *Int. J. Plant Res.* 28 82–89. 10.5958/2229-4473.2015.00041.5

[B18] Lai Z. Lin Y. (2013). Analysis of global transcriptome of longan (*Dimocarpus longan* Lour.) embryogenic callus using illumina paired-end sequencing. *BMC Genomics* 14:561. 10.1186/1471-2164-14-561 23957614 PMC3765095

[B19] Mckain M. R. Wickett N. Zhang Y. Ayyampalayam S. McCombie W. R. Chase M. W. (2012). Phylogenomic analysis of transcriptome data elucidates co-occurrence of a paleopolyploid event and the origin of bimodal karyotypes in Agavoideae (Asparagaceae). *Am. J. Bot.* 99 397–406. 10.3732/ajb.1100537 22301890

[B20] Mooney S. (2005). Bioinformatics approaches and resources for single nucleotide polymorphism functional analysis. *Brief. Bioinform.* 6 44–56. 10.1093/bib/6.1.44 15826356

[B21] Morikawa T. Mizutani M. Ohta D. (2006). Cytochrome P450 subfamily CYP710A genes encode sterol C-22 desaturase in plants. *Biochem. Soc. Trans.* 34 1202–1205. 10.1042/BST0341202 17073785

[B22] Murray M. Thompson W. F. (1980). Rapid isolation of high molecular weight plant DNA. *Nucleic Acids Res.* 8 4321–4325. 10.1093/nar/8.19.4321 7433111 PMC324241

[B23] Ophir R. Sherman A. Rubinstein M. Eshed R. Schwager M. S. Harelbeja R. (2014). Single-nucleotide polymorphism markers from de-novo assembly of the pomegranate transcriptome reveal germplasm genetic diversity. *PLoS One* 9:e88998. 10.1371/journal.pone.0088998 24558460 PMC3928336

[B24] Pavy N. Deschênes A. Blais S. Lavigne P. Beaulieu J. Isabel N. (2013). The landscape of nucleotide polymorphism among 13,500 genes of the conifer *Picea glauca*, relationships with functions, and comparison with *Medicago truncatula*. *Genome Biol. Evol.* 5 1910–1925. 10.1093/gbe/evt143 24065735 PMC3814201

[B25] Pons O. Petit R. J. (1996). Measuring and testing genetic differentiation with ordered versus unordered alleles. *Genetics* 144 1237–1245. 8913764 10.1093/genetics/144.3.1237 PMC1207615

[B26] Rozas J. Sánchez-DelBarrio J. C. Messeguer X. Rozas R. (2003). DnaSP, DNA polymorphism analyses by the coalescent and other methods. *Bioinformatics* 19 2496–2497. 10.1093/bioinformatics/btg359 14668244

[B27] Sambrook J. Russell D. (2001). *Molecular Cloning, a Laboratory Manual.* New York, NY: Cold Spring Harbor Laboratory Press.

[B28] Schalk M. Pierrel M. A. Zimmerlin A. (1997). Xenobiotics: substrates and inhibitors of the plant cytochrome P450. *Environ. Sci. Pollut. Res. Int.* 4 229–234. 10.1007/BF02986353 19005807

[B29] Shih C. (1979). *Opisthopappus shih*-a new genus of compositae from China. *Acta Phytotaxon. Sin.* 32 110–112.

[B30] Shih C. Fu G. X. (1983). *Angiospermae, Dicotyledoneae, Compositae (3) Anthemideae, Angiospermae. Flora Republicae Popularis Sinicae, China.* Beijing: Science Press.

[B31] Tamura K. Dudley J. Nei M. Kumar S. (2007). MEGA: molecular evolutionary genetics analysis (MEGA) software. *Mol. Biol. Evol.* 24 1596–1599. 10.1093/molbev/msm092 17488738

[B32] Tang F. P. Wang H. B. Chen S. M. Chen F. Teng N. Liu Z. (2012). First intergeneric hybrids within the tribe Anthemideae cass. III. *Chrysanthemum indicum* L. Des *Moul. × Opisthopappus* taihangensis (Ling) shih. *Biochem. Syst. Ecol.* 43 87–92. 10.1016/j.bse.2012.02.007

[B33] Thomas P. D. Kejariwal A. (2004). Coding single-nucleotide polymorphisms associated with complex vs. Mendelian disease: evolutionary evidence for differences in molecular effects. *Proc. Natl. Acad. Sci. U.S.A.* 101 15398–15403. 10.1073/pnas.0404380101 15492219 PMC523449

[B34] Thompson J. D. Gibson T. J. Plewniak F. Jeanmougin F. Higgins D. G. (1997). The ClustalX windows interface: flexible strategies for multiple sequence alignment aided by quality analysis tools. *Nucleic Acids Res.* 25 4876–4882. 10.1093/nar/25.24.4876 9396791 PMC147148

[B35] Tong C. Wang X. Yu J. Wu J. Li W. Huang J. (2013). Comprehensive analysis of RNA-seq data reveals the complexity of the transcriptome in *Brassica rapa*. *BMC Genomics* 14:689. 10.1186/1471-2164-14-689 24098974 PMC3853194

[B36] Wang F. Z. Tang J. Chen X. Q. Liang S. J. Dai L. K. (1978). *Liliaceae. Flora of China Editorial Committee of China Academy of Science.* Beijing: Science Press, 73–74.

[B37] Wang S. Xie Y. (2009). *China Species Red List.* Beijing: Higher Education Press.

[B38] Wang Y. Xu L. Chen Y. Shen H. Gong Y. Limera C. (2013). Transcriptome profiling of radish (*Raphanus sativus* L.) root and identification of genes involved in response to lead (Pb) stress with next generation sequencing. *PLoS One* 8:e66539. 10.1371/journal.pone.0066539 23840502 PMC3688795

[B39] Wang Y. L. (2013). Chloroplast microsatellite diversity of *Opisthopappus shih* (Asteraceae) endemic to China. *Plant Syst. Evol.* 299 1849–1858. 10.1007/s00606-013-0840-8

[B40] Wang Y. L. Yan G. Q. (2013). Genetic diversity and population structure of *Opisthopappus longilobus* and *Opisthopappus taihangensis* (Asteraceae) in China determined using sequence related amplified polymorphism markers. *Biochem. Syst. Ecol.* 49 115–124. 10.1016/j.bse.2013.03.014

[B41] Wang Y. L. Yan G. Q. (2014). Molecular phylogeography and population genetic structure of *O. longilobus* and *O. taihangensis (Opisthopappus)* on the Taihang Mountains. *PLoS One* 9:e104773. 10.1371/journal.pone.0104773 25148249 PMC4141751

[B42] Wang Y. L. Zhang C. Q. Lin L. L. Yuan L. H. (2015). ITS sequence analysis of *Opisthopappus taihangensis* and *Opisthopappus longilobus*. *Acta Hortic. Sin.* 42 178–186.

[B43] Wei L. Li S. Liu S. He A. Wang D. Wang J. (2014). Transcriptome analysis of *Houttuynia cordata* Thunb. by Illumina paired-end RNA sequencing and SSR marker discovery. *PLoS One* 9:e84105. 10.1371/journal.pone.0084105 24392108 PMC3879290

[B44] Wen J. Xiong Z. Nie Z. L. Mao L. Zhu Y. Kan X. Z. (2013). Transcriptome sequences resolve deep relationships of the grape family. *PLoS One* 8:e74394. 10.1371/journal.pone.0074394 24069307 PMC3775763

[B45] Xu W. B. Guo Q. S. Wang C. L. (2006). RAPD analysis for genetic diversity of *Chrysanthemum morifolium*. *Chin. J. Chin. Mat. Med.* 21 1645–1648. 16548159

[B46] Xu X. Pan S. Cheng S. Zhang B. Mu D. Ni P. (2011). Genome sequence and analysis of the tuber crop potato. *Nature* 475 189–195. 10.1038/nature10158 21743474

[B47] Xue S. Liu Y. Zhang Y. Sun Y. Geng X. Sun J. (2013). Sequencing and de novo analysis of the hemocytes transcriptome in *Litopenaeus vannamei* response to white spot syndrome virus infection. *PLoS One* 8:e76718. 10.1371/journal.pone.0076718 24204661 PMC3799976

[B48] Yang D. Y. Hu X. Liu Z. H. Zhao H. (2010). Intergeneric hybridizations between *Opisthopappus taihangensis* and *Chrysanthemum lavandulifolium*. *Sci. Hortic.* 125 718–723. 10.1016/j.scienta.2010.05.002

[B49] Ye Q. Qiang-Hua X. U. (2012). The prokaryotic expression of recombinant heat shock protein HSP90a of Portunus trituberculatus under salinity stress. *Iran. J. Fish. Sci.* 36:681.

[B50] Zhao H. B. Chen F. D. Chen S. M. Wu G. Guo W. (2010). Molecular phylogeny of *Chrysanthemum*, Ajania and its allies (Anthemideae, Asteraceae) as inferred from nuclear ribosomal ITS and chloroplast trnL-F IGS sequences. *Plant Syst. Evol.* 284 153–169. 10.1007/s00606-009-0242-0

[B51] Zhao Z. Wu G. Wang J. Liu C. Qiu L. (2013). Next-generation sequencing-based transcriptome analysis of Helicoverpa armigera larvae immune-primed with *Photorhabdus luminescens* TT01. *PLoS One* 8:e80146. 10.1371/journal.pone.0080146 24302999 PMC3841171

