# Supplementary material for: De novo Assembly and Transcriptome Characterization of Opisthopappus (Asteraceae) for Population Differentiation and Adaption
Source: Front Genet. 2018 Sep 19;9:371. doi: 10.3389/fgene.2018.00371 (PMC6156141; doi:10.3389/fgene.2018.00371)
Supplement: Supplementary file 1 [file Table_1.doc]

# Table S1. Information of SNP primer pairs

| Primer name | Primers sequences 5′-3′ | AT（℃） |
| --- | --- | --- |
| SNP1 | A: CAACTTGTTAGGGTCATCCA  S: TGTCCGAGGTTTATTTAGGG | 53 |
| SNP2 | A: GGCAACCATGATCCACCAGC  S:GGACGGATTCATCGGTAGGG | 61 |
| SNP3 | A: CACTCAATCGCACTCCTTTC  S:CGCTCGTAGTAATCGCAGAC | 55 |
| SNP4 | A: TGTTCAACGTCATCGCCCTA S:CCCCTGATAAAGTCGCCTCG | 60 |
| SNP5 | A:AACCCAACAGGATCATTCG S:ATCACTCATTTCCGCAAACCTAA | 55 |
| SNP6 | A: CAAGTTCCTCCTCAAATCCC S:TGTCCGTCACCGTTACCCTA | 56 |
| SNP7 | A:CATTGATGGTCGTTAGGTTTG S:AATACTTGAGCCGAGTTTGC | 53 |
| SNP8 | A:TGGTGACTACGGGTGGGACA S:CCTGAACAAAGAAACCGAACA | 54 |

Table S2. The statistical analysis of functional annotation of unigenes against different databases

| Database | Number | Percentage |
| --- | --- | --- |
| NR | 33975 | 100 |
| GO | 33975 | 100 |
| KEGG | 4857 | 14.3 |
| eggNOG | 10357 | 30.48 |
| Swissprot | 30789 | 90.62 |
| In all database | 990 | 2.91 |

Abbreviations: NR, NCBI non-redundant protein sequences; GO, Gene Ontology;

KEGG, Kyoto Encyclopedia of Genes and Genome; eggNOG, evolutionary

genealogy of genes Non-supervised Orthologous Groups.

Table S3. The number of significant differentially regulated unigenes

| Case | Control | Up-regulated unigene | | Down-regulated unigene | | Total  DE unigene | |
| --- | --- | --- | --- | --- | --- | --- | --- |
| *Opisthopappus longilobu* ^a^ | *Opisthopappus taihangensis*^b^ | Number | % | Number | % | Number | % |
|  |  | 1925 | 5.66 | 1485 | 4.37 | 3410 | 10.04 |

a Within the column standan for *Opisthopappus longilobus*;

b Within the column standan for *Opisthopappus taihangensis*.

Table S4. Population pairwise *F*_ST_s of *Opisthopappus* Shih.

|  | HDX | BXT | JNH | LFS | XT | SBY | WWS | LQZ | XYG | SNS | BQ | QLX |
| --- | --- | --- | --- | --- | --- | --- | --- | --- | --- | --- | --- | --- |
| HDX | 0.00000 |  |  |  |  |  |  |  |  |  |  |  |
| BXT | 0.20213 | 0.00000 |  |  |  |  |  |  |  |  |  |  |
| JNH | 0.17197 | 0.07812 | 0.00000 |  |  |  |  |  |  |  |  |  |
| LFS | 0.34593 | 0.26554 | 0.16667 | 0.00000 |  |  |  |  |  |  |  |  |
| XT | 0.38889 | 0.18033 | 0.16071 | 0.48187 | 0.00000 |  |  |  |  |  |  |  |
| SBY | 0.07169 | 0.10156 | 0.08898 | 0.01232 | 0.33480 | 0.00000 |  |  |  |  |  |  |
| WWS | 0.86815 | 0.82240 | 0.79041 | 0.83765 | 0.90318 | 0.78784 | 0.00000 |  |  |  |  |  |
| LQZ | 0.84091 | 0.81081 | 0.79412 | 0.82787 | 0.86486 | 0.78795 | 0.38815 | 0.00000 |  |  |  |  |
| XYG | 0.87719 | 0.84783 | 0.82927 | 0.86111 | 0.90000 | 0.82543 | 0.60177 | 0.11607 | 0.00000 |  |  |  |
| SNS | 0.83411 | 0.80324 | 0.78664 | 0.82143 | 0.85880 | 0.77982 | 0.51075 | 0.01562 | 0.16118 | 0.00000 |  |  |
| BQ | 0.87395 | 0.84583 | 0.82812 | 0.85878 | 0.89583 | 0.82438 | 0.67789 | 0.14557 | 0.20635 | 0.29847 | 0.00000 |  |
| QLX | 0.89823 | 0.86842 | 0.84836 | 0.88000 | 0.92105 | 0.84565 | 0.74359 | 0.09091 | 0.07143 | 0.29795 | 0.50658 | 0.00000 |

Figure S1. Size distribution of the transcript

The abundance of transcript based on nucleotide length (nt) in the size range of 100–500, 500–1,000, 1,000–1,500, 1500–2,000 and more than 2,000 nt.

Figure S2. Size distribution of the assembled 33975 unigenes

The abundance of unigenes assembled based on nucleotide length (nt) in the size range of 100–500, 500–1,000, 1,000–1,500, 1500–2,000 and more than 2,000 nt.

Figure S3. Histogram presentation of Clusters of Orthologus Groups classification of 10357 known protein annotated unigenes

Each bar represents the number of unigenes classified into each of the 24 COG functional categories.

Figure S4. KEGG biological pathway classification histograms for 4857 protein annotated unigenes

Each bar represents the number of unigenes that are systematically categorized into sub-classes under Metabolism, Genetic Information Processing, Environmental Information Processing, Cellular Processes, Organismal Systems and Human Diseases.

Figure S5. The estimated best genetic structure value of *K* based on structure harvest online
