# Supplementary figures and images for: De novo Assembly and Transcriptome Characterization of Opisthopappus (Asteraceae) for Population Differentiation and Adaption
Source: Front Genet. 2018 Sep 19;9:371. doi: 10.3389/fgene.2018.00371 (PMC6156141; doi:10.3389/fgene.2018.00371)

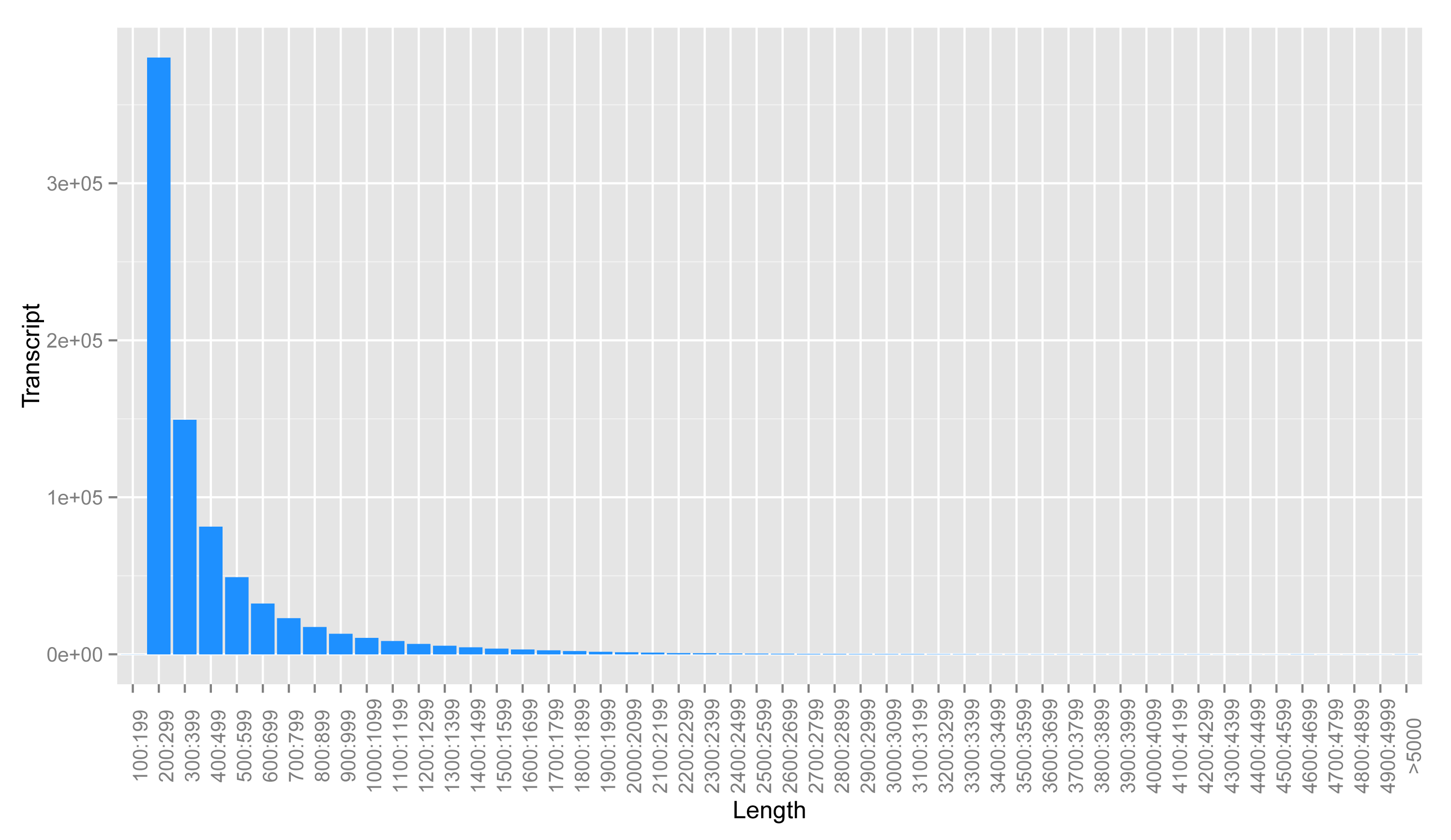

Supplement: Supplementary file 2 [file Image_1.TIF]

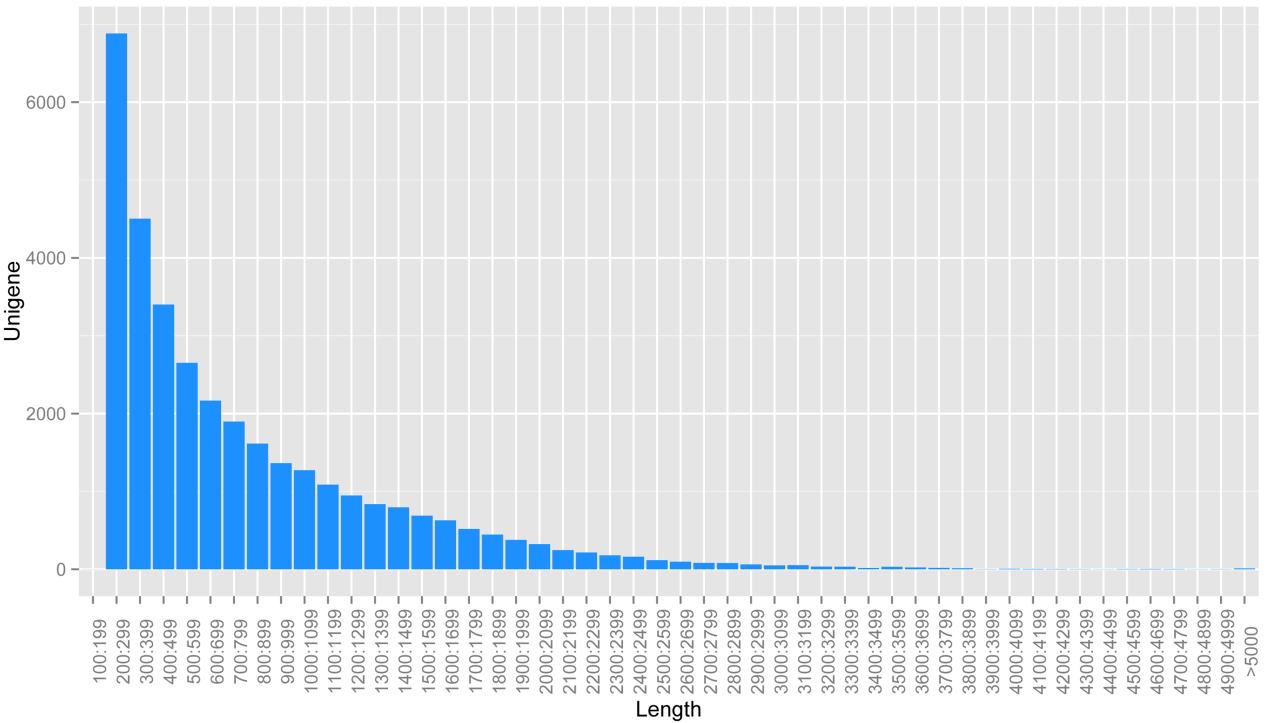

Supplement: Supplementary file 3 [file Image_2.TIF]

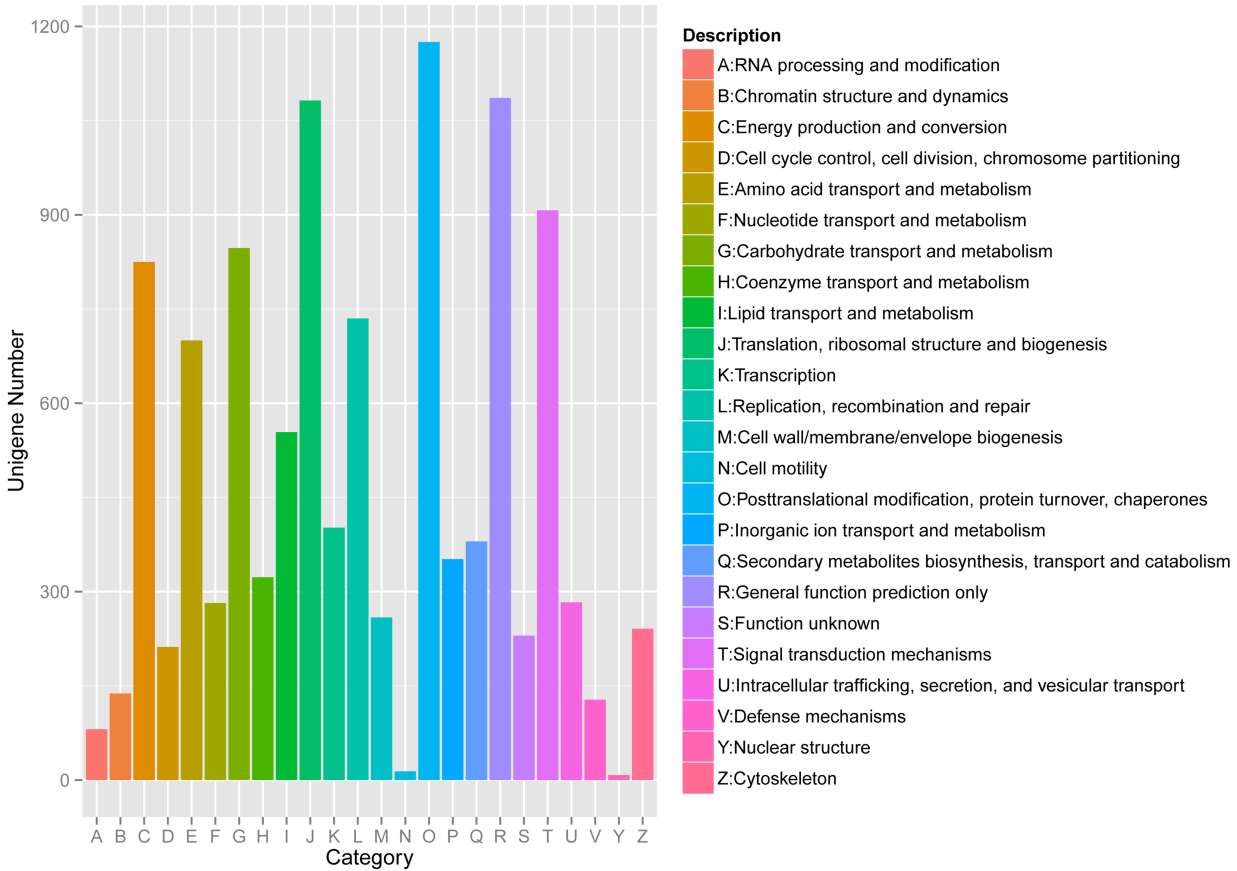

Supplement: Supplementary file 4 [file Image_3.TIF]

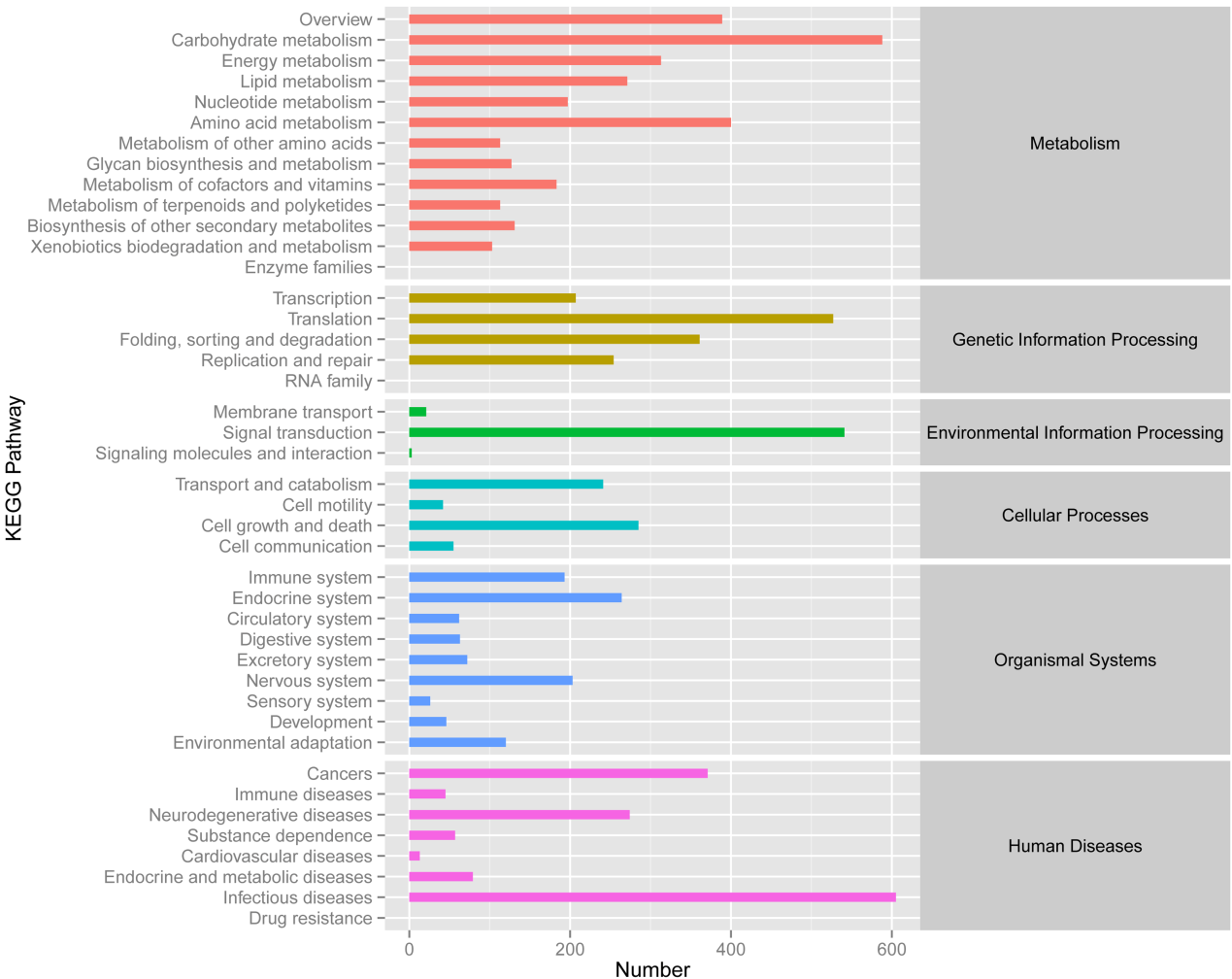

Supplement: Supplementary file 5 [file Image_4.TIF]
